# No Association between 2008–09 Influenza Vaccine and Influenza A(H1N1)pdm09 Virus Infection, Manitoba, Canada, 2009

**DOI:** 10.3201/eid1805.111596

**Published:** 2012-05

**Authors:** Salaheddin M. Mahmud, Paul Van Caeseele, Gregory Hammond, Carol Kurbis, Tim Hilderman, Lawrence Elliott

**Affiliations:** University of Manitoba, Winnipeg, Manitoba, Canada (S.M. Mahmud, P. Van Caeseele, G. Hammond, T. Hilderman, L. Elliott);; Winnipeg Regional Health Authority, Winnipeg (S.M. Mahmud, C. Kurbis);; Cadham Provincial Laboratory, Winnipeg (P.V. Caeseele);; Manitoba Health, Winnipeg (T. Hilderman, L. Elliott)

**Keywords:** epidemiology, pandemic (H1N1) 2009, pandemic (H1N1) 2009 virus, pandemic A(H1N1)2009 virus, influenza A(H1N1)pdm09, trivalent influenza vaccine, 2008–09 inactivated trivalent influenza vaccine, TIV, case–control study, influenza, Canada, viruses

## Abstract

We conducted a population-based study in Manitoba, Canada, to investigate whether use of inactivated trivalent influenza vaccine (TIV) during the 2008–09 influenza season was associated with subsequent infection with influenza A(H1N1)pdm09 virus during the first wave of the 2009 pandemic. Data were obtained from a provincewide population-based immunization registry and laboratory-based influenza surveillance system. The test-negative case–control study included 831 case-patients with confirmed influenza A(H1N1)pdm09 virus infection and 2,479 controls, participants with test results negative for influenza A and B viruses. For the association of TIV receipt with influenza A(H1N1)pdm09 virus infection, the fully adjusted odds ratio was 1.0 (95% CI 0.7–1.4). Among case-patients, receipt of 2008–09 TIV was associated with a statistically nonsignificant 49% reduction in risk for hospitalization. In agreement with study findings outside Canada, our study in Manitoba indicates that the 2008–09 TIV neither increased nor decreased the risk for infection with influenza A(H1N1)pdm09 virus.

## Abstract 2

Receipt of seasonal inactivated trivalent vaccine neither increased nor decreased the risk for pandemic influenza virus infection.

The nature of the relationship between receipt of the 2008–09 seasonal inactivated trivalent influenza vaccine (TIV) and the risk for infection with the pandemic (H1N1) 2009 virus strain, hereafter referred to as A(H1N1)pdm09, remains unclear. A case–control study in Canada that used data from a network of sentinel physicians monitoring influenza vaccine effectiveness in the provinces of Alberta, British Colombia, Ontario, and Quebec found an increased risk for influenza A(H1N1)pdm09 virus infection among persons who received the 2008–09 TIV (odds ratio [OR] 1.7, 95% CI 1.0–2.7); an increased risk for severe illness was not detected (*1*). In addition, 3 other studies conducted by the same team found a 1.4- to 2.5-fold increased risk for infection (laboratory confirmed) with influenza A(H1N1)pdm09 virus among persons who received the 2008–09 TIV (*1*). The 3 studies were 1) a test-negative case–control study in Ontario; 2) a household transmission cohort study in Quebec; and 3) a conventional case–control study using population controls in Quebec.

The results of these studies in Canada were not confirmed by studies conducted elsewhere. In fact, several studies using different designs found that TIV partially prevented or had no effect on infections with the pandemic strain (*2**–**16*). It has been suggested that the finding in Canada of an increased risk for influenza A(H1N1)pdm09 virus infection among persons who received the 2008–09 TIV might be unique to Canada; the increased risk might be related to the use of the domestically manufactured vaccine (*1*) or to the timing of the pandemic in relation to the most recent influenza season and the types of circulating influenza strains during that season (*17**,**18*). At the time of the pandemic, the Canadian province of Manitoba was not part of the Canadian vaccine effectiveness monitoring network. However, the availability of a provincewide, population-based immunization registry and laboratory-based influenza surveillance system provided a unique opportunity to investigate these issues in Manitoba.

In the first wave of the pandemic (May–August 2009), Manitoba was more severely affected than any other Canadian province, accounting for 50% of hospital intensive care unit admissions attributable to the virus in Canada (*19**,**20*). TIVs used in Manitoba during the 2008–09 influenza season were identical to those used elsewhere in Canada; they included 15 μg hemagglutinin each of A/Brisbane/59/2007 (H1N1)–like virus, A/Brisbane/10/2007 (H3N2)–like virus, and B/Florida/4/2006-like virus. These were the 3 strains recommended that year by the World Health Organization for influenza vaccines in the Northern and Southern Hemispheres (*21*). In Manitoba, as in other provinces, ≈75% of the administered seasonal influenza vaccine doses were manufactured domestically (Fluviral; GlaxoSmithKline, Mississauga, Ontario, Canada); imported vaccines, predominantly Vaxigrip (Sanofi Pasteur Ltd, Toronto, Ontario, Canada), comprised the remaining 25%. The live attenuated influenza vaccine was not available in Canada during the 2008–09 season.

To investigate whether use of TIV in Manitoba was associated with influenza A(H1N1)pdm09 virus infection during the first wave of the pandemic, we conducted a population-based case–control study using data from Cadham Provincial Laboratory (CPL) and the Manitoba Immunization Monitoring System. The test-negative case–control design used in this study is similar to the design of the Ontario study (*1*).

## Methods

### Data Sources

This study was conducted using de-identified records obtained by linking the CPL database, the Manitoba Immunization Monitoring System, and other Manitoba Health (MH) administrative databases after securing the approval of the Research Ethics Board of the University of Manitoba and the Health Information Privacy Committee of MH. MH provides publicly funded health insurance coverage to 99% of the 1.2 million residents of Manitoba. The coverage includes laboratory testing and hospital and outpatient physician services, including immunization and laboratory services. Eligibility for coverage is not based on age or income. For administrative purposes, MH maintains several centralized electronic databases that can be linked by using a unique health services number.

### Study Population

All Manitoba residents >6 months of age who had respiratory specimens submitted to CPL, the province’s only public health laboratory, for influenza testing during April 27–August 21, 2009, were included in this analysis. During the pandemic, guidelines for testing patients seeking care for influenza-like illness were issued by MH; anecdotal evidence indicated that, to a great extent, physicians followed the guidelines. Patients were tested in hospital and ambulatory care settings; the specimens obtained were predominantly nasopharyngeal and nasal swab samples. For the duration of the first pandemic wave, all influenza testing in Manitoba was completed at CPL by using a real-time duplex reverse transcription PCR (RT-PCR) developed by the National Microbiology Laboratory (*22*). We obtained information about influenza testing from CPL’s electronic database.

### Study Design

Consistent with the design of the Ontario test-negative case–control study (*1*), we identified 3 nonoverlapping study groups: 1) the hospitalized cases group comprised persons in the study population (as defined above) who had real-time duplex RT-PCR test results positive for influenza A(H1N1)pdm09 virus and who had been admitted to a hospital in Manitoba around the time of testing (within ± 1 week of collection of their first influenza A(H1N1)pdm09 virus–positive specimen); 2) the community cases group comprised A(H1N1)pdm09-positive persons who had not been hospitalized during April–August 31, 2009; 3) the community controls group comprised persons who were not hospitalized during this period and who tested negative for influenza A and B. Identification of these 3 groups enabled us to assess whether use of TIV was associated with the detection of influenza A(H1N1)pdm09 virus infection (by contrasting the odds of TIV use among community case-patients and community controls). Identification of the 3 groups also enabled us to assess whether use of TIV was associated with increased risk for hospitalization as an indication of the severity of disease (by contrasting the odds of TIV use among hospitalized and community case-patients). Information about hospitalization status was obtained from the Hospital Separation database.

### Determination of Vaccination Status

For all study participants, information about receipt of TIV and polyvalent pneumococcal polysaccharide (PPV23) vaccine during or before the 2009–10 influenza season was obtained from the Manitoba Immunization Monitoring System, the population-based provincewide registry that has recorded virtually all vaccinations administered to Manitobans since 1988. In addition to details about patients, the database stores information about the date of vaccination and the type and dose, but not the brand name, of the vaccine administered. The recorded vaccination information is considered highly complete and accurate (*23*).

### Information about Potential Confounders

Study participants were assigned to a neighborhood of residence based on their postal code as recorded in the MH Population Registry. Information about socioeconomic status was obtained by using the postal code of residence and a previously validated area-based Socioeconomic Factor Index (SEFI) (*24*).

Information about coexisting diseases and propensity to seek health care (measured as the number of hospital and family physician visits in the previous 12 months) was obtained from the Hospital Separation and Physician Claims databases. Since 1971, these databases have recorded information about most hospital admissions and outpatient physician visits, respectively. Previously validated algorithms were used to identify various chronic diseases and other indications for vaccination (*25*) (Table 1). Immunosuppression was defined as having a diagnosis of cancer, AIDS, or another immunodeficiency disorder or as receiving prescriptions for immunosuppressive drugs. Information about the use of immunosuppressant and antimicrobial drugs and neuraminidase inhibitors was obtained from the Drug Program Information Network, the comprehensive database of all out-of-hospital prescriptions dispensed in Manitoba. Pregnancy status was determined from the databases mentioned above by using disease and tariff codes for different conditions and procedures indicative of ongoing pregnancy or the completion of pregnancy (*26*) (Table 1).

**Table 1:** Definitions of variables used in analyses to determine possible association between receipt of 2008–09 TIV and subsequent infection with influenza A(H1N1)pdm09 virus, Manitoba, Canada, 2009*†

Variable	Definition
Drugs‡	
For HIV	Protease inhibitors (J05AE*), nucleoside and nucleotide reverse transcriptase inhibitors (J05AF*), nonnucleoside reverse transcriptase inhibitors (J05AG*), antivirals for treatment of HIV infections, combinations (J05AR*)
For influenza	Neuraminidase inhibitors (J05AH*) or cyclic amines (J05AC*)
For diabetes	Drugs used in diabetes (A10*), insulins and analogs (A10A*), blood glucose lowering drugs, excluding insulins (A10B*)
Immunosuppressants	Antineoplastic agents (L01*), immunosuppressants (L04A*)
Systemic antimicrobials	Antibacterials for systemic use (J01*), antimycotics for systemic use (J02*), antimycobacterials (J04*)
Systemic steroids	Corticosteroids for systemic use, plain (H02A*), glucocorticoids (H02AB*), corticosteroids for systemic use, combinations (H02B*)
Pregnancy§	
Ongoing pregnancy	>1 admission code (O10–16, O20–29, O30–48, O94–99, Z32–36) or >2physician claims (640–649, V22) or >1 tariff code for prenatal services; must be within ±30 d of the index date (*26*)
Completion of pregnancy	>1 admission code (O8, O65–75, O80–84, O85–92, Z37–39) or >2 physician claims (650–659, 670–676) or >1 tariff code for delivery, abortion or postnatal services; must be within 270 d following the index date (*26*)
Medical condition§¶	
Alcoholism	>1 admission (E24.4, E51.2, E52, F10, G31.2, G62.1, G72.1, I42.6, K29.2, K70, K86.0, O35.4, P04.3, Q86.0, R78.0, T50.6, T51.0, T51.1, T51.9, X45, X65, Y15, Y57.3, Y90, Y91, Z50.2, Z71.4, Z72.1, Z81.1) or >2 physician claims (291, 303)
Anemia	>1 admission (D50–64) or >2 physician claims (280–285)
Asthma	>1 admission (J45, J46) or >2 physician claims (493)
Cancer, excluding NMS	>1 admission (C00–43, C45–97) or >1 physician claim (140–172, 174–209, 235–239)
Cardiovascular disease	>1 admission (I00–99, O11) or >2 physician claims (390–519)
Chronic renal failure	>1 admission (I12.0, I13.1, N18, N19, N25.0, Z49, Z99.2) or >2 physician claims (403–404, 586–587)
Chronic respiratory condition	>1 admission (J40–99, O24) or >2 physician claims (490–496, 500–508)
COPD	>1 admission (J40–44, O24) or >2 physician claims (630–633, 634–639, 490–492, 496)
Diabetes	>1 admission (E10–14, G590, G632, H280, H360, M142, M146, N083, O24) or >2 physician claims (250) or >2 prescriptions for drugs used in treatment of diabetes
HIV/AIDS	>1 admission (B20–24, R75, Z21) or >2 physician claims (042 V08) or >1 prescriptions for drugs used in treatment of HIV
Hypertension	>1 admission (I10–15, I67.4, O11) >2 physician claims (401–405)
Immunodeficiency	>1 admission (D80–D84, D89) or >2 physician claims (288, 279)
Immunosuppressed	Having an organ transplant or a diagnosis of HIV/AIDS, other immunodeficiency disorders or cancer (other than nonmelanoma skin cancer), or receiving prescriptions for immunosuppressants or systemic steroids
Ischemic heart disease	>1 admission (I20–25) or >1 physician claims (410–414)
Organ transplant	>1 admission (T86, Y83.0, Z94) or >2 physician claims (V42)
Stroke	>1 admission (I61, I63, I64, I67.9, I69) or >2 physician claims (431, 434, 436–438)
Substance abuse	>1 admission (F11–16, F18–19) or >2 physician claims (292, 304,305)
Other	
Area of residence	Based on postal code of current address and the North-South relationship variable derived by using PCCF+#; North includes areas coded as “North” or "North transition"; South includes areas coded as "South" or "South transition"

*Asterisks indicate a wild-card character used as an alternative to listing all disease or drug codes within a section or subsection of the corresponding classification system. †TIV, inactivated trivalent influenza vaccine; NMS, nonmelanoma skin cancer; COPD, chronic obstructive pulmonary disease. ‡Drugs were classified based according to their drug identification number and the Anatomical Therapeutic Chemical Classification System with Defined Daily Doses (www.who.int/classifications/atcddd/en/). §Codes in parentheses are International Classification of Diseases (ICD)-10-CA codes for hospital admission data and ICD-9-CM codes for physician claims data. ¶Based on previously validated chronic disease identification algorithms with modifications. #Automated Postal Code Conversion File Plus system, Statistics Canada (www.statcan.gc.ca/bsolc/olc-cel/olc-cel?lang=eng&catno=82F0086X).

### Statistical Analysis

We used unconditional logistic regression models (fitted to community case-patients and community controls) to estimate odds ratios (ORs) for the association between the receipt of the 2008–09 TIV and subsequent infection with laboratory-confirmed influenza A(H1N1)pdm09 virus while adjusting for confounding. Results are presented for unadjusted models (model A) and for models that were adjusted a priori for age, sex, place of residence, SEFI, and week of specimen collection (to account for changes in infection incidence and laboratory testing practices) (model B). Other potential confounders (Table 1) were included in the fully adjusted models if their inclusion resulted in a >2% change in crude ORs. Using this criterion, we also adjusted the final models (model C) for pregnancy, antiviral drug use, presence of a chronic or immunocompromising medical condition, and number of hospital admissions and family physician visits in the previous 12 months. Model C also included mutual adjustment for the 2007–08 TIV and the PPV23.

These analyses were repeated after stratification by potential confounders and effect modifiers, such as age group, place of residence, epidemic phase (before and after the peak), and presence of chronic conditions. We also assessed for possible effect modification between the 2008–09 TIV and the 2007–08 TIV and the PPV23. The statistical significance of adding the interaction terms was assessed by using a likelihood ratio test. Similar analyses, contrasting the odds of TIV use among hospitalized case-patients with those among community case-patients, were performed to assess whether use of the 2008–09 TIV was associated with increased risk for hospitalization.

## Results

During the study period, 4,275 persons were tested for influenza. Of them, 879 (20.6%) were positive for influenza A(H1N1)pdm09 virus, 3,391 (79.3%) were negative for all influenza viruses, and 5 who were positive for influenza A but negative for the pandemic virus were excluded from study. We also excluded 35 persons (8 case-patients, 27 controls) who did not usually reside in Manitoba and 185 infants (26 case-patients, 159 controls) who were <6 months of age. A total of 726 hospitalized test-negative controls and 14 case-patients who were hospitalized during the study period but not around the time of testing were also excluded. Thus, there was a total of 3,310 study participants: 205 hospitalized case-patients, 626 community case-patients, and 2,479 community test-negative controls.

Consistent with previous reports from Manitoba (*19**,**27*), we found that the influenza A(H1N1)pdm09 virus–positive case-patients during the first pandemic wave were younger and more socioeconomically disadvantaged than controls (Table 2). Probably because they were younger, community case-patients had fewer prior hospitalizations and physician visits and were less likely than controls to have had a diagnosed chronic or immunocompromising medical condition. Consistent with the literature (*20**,**27*), we also found that younger children, pregnant women, residents of northern Manitoba, socioeconomically disadvantaged persons, and persons with chronic diseases were more likely to be hospitalized for infection with the pandemic virus.

**Table 2:** Demographic and clinical characteristics of persons enrolled in a study to determine possible association between receipt of 2008–09 TIV and subsequent infection with influenza A(H1N1)pdm09 virus, Manitoba, Canada, 2009*

Characteristic	Community controls	Community case-patients	Hospitalized case-patients	Total
Total	2,479 (74.9)	626 (18.9)	205 (6.2)	3,310
Female sex	1,468 (59.2)	322 (51.4)	115 (56.1)	1,905
Age, y				
0.5–4	210 (8.5)	62 (9.9)	48 (23.4)	320
5–19	413 (16.7)	223 (35.6)	41 (20.0)	677
20–34	553 (22.3)	154 (24.6)	44 (21.5)	751
35–49	600 (24.2)	110 (17.6)	35 (17.1)	745
50–59	331 (13.4)	56 (8.9)	20 (9.8)	407
>60	372 (15.0)	21 (3.4)	17 (8.3)	410
Age, y, median (Q1–Q3)	36 (19–51)	22 (10–39)	23 (5–43)	34 (16–52)
Residence				
Northern Manitoba	362 (14.6)	156 (24.9)	82 (40.0)	600
Urban area	1,406 (56.7)	341 (54.5)	88 (42.9)	1835
SEFI quintile†				
1	596 (24.0)	106 (16.9)	22 (10.7)	724
2	541 (21.8)	140 (22.4)	28 (13.7)	709
3	499 (20.1)	126 (20.1)	25 (12.2)	650
4	468 (18.9)	130 (20.8)	41 (20.0)	639
5	375 (15.1)	124 (19.8)	89 (43.4)	588
Physician visits in past year, median (Q1–Q3)	14 (5–32)	7 (3–19)	20 (10–51)	15 (6–34)
Hospitalizations in past 5 y, median (Q1–Q3)	0 (0–1)	0 (0–1)	3 (1–5)	1 (0–2)
Pregnant‡	85 (5.8)	17 (5.3)	25 (21.7)	127
Chronic disease	548 (22.1)	90 (14.4)	78 (38.0)	716
Diabetes	219 (8.8)	30 (4.8)	35 (17.1)	284
COPD	98 (4.0)	11 (1.8)	15 (7.3	124
Asthma	133 (5.4)	38 (6.1)	25 (12.2)	196
Ischemic heart disease	51 (2.1)	6 (1.0)	6 (2.9)	63
Chronic renal failure	43 (1.7)	5 (0.8)	10 (4.9)	58
Cancer, excluding NMS	95 (3.8)	7 (1.1)	10 (4.9)	112
Receipt of drug treatment				
Antiviral drug treatment	276 (11.1)	123 (19.6)	15 (7.3)	414
Antiviral prophylaxis	72 (2.9)	22 (3.5)	3 (1.5)	97
Antimicrobial drug treatment	1,035 (41.8)	219 (35.0)	106 (51.7)	1,360
Receipt of TIV				
2007–08	544 (21.9)	123 (19.6)	37 (18.0)	704
2008–09	564 (22.8)	109 (17.4)	39 (19.0)	712
No. TIVs received in past 5 years				
0	1,450 (58.5)	383 (61.2)	119 (58.0)	1,952
>1	1,029 (41.5)	243 (38.8)	86 (42.0)	1,358
1–3	711 (28.7)	187 (29.9)	65 (31.7)	963
4–5	318 (12.8)	56 (8.9)	21 (10.2)	395
5	156 (6.3)	19 (3.0)	9 (4.4)	184
Ever received PPV23	343 (13.8)	41 (6.5)	32 (15.6)	416

*Values are no. (%) except as indicated. See Table 1 for definitions of variables. TIV, inactivated trivalent influenza vaccine; Q1–Q3, quartiles 1-3; SEFI, Socioeconomic Factor Index; COPD, chronic obstructive pulmonary disease; NMS, nonmelanoma skin cancer; PPV23, pneumococcal polysaccharide vaccine. †SEFI quintiles are in order of worsening socioeconomic scale (*24*). ‡Of 5- to 49-year-old female participants.

About 17% of the community case-patients and 23% of the community controls received TIV during the 2008–09 influenza season (Table 3). The crude OR for the association of TIV receipt with subsequent infection with influenza A(H1N1)pdm09 virus was 0.7 (95% CI 0.6–0.9), corresponding to a vaccine effectiveness estimate of 30%. Adjusting for age, sex, region of residence, SEFI, and week of specimen collection (model B) resulted in an OR of 1.1 (95% CI 0.8–1.4). Additional adjustment for all other measured confounders (model C) did not appreciably change the OR estimates (OR 1.0, 95% CI 0.7–1.4).

**Table 3:** Association between receipt of seasonal influenza vaccine and subsequent infection with influenza A(H1N1)pdm09 virus, by vaccine type, Manitoba, Canada, 2009*

Variable	No. community controls, n = 2,479	No. community case-patients, n = 626	Odds ratio (95% CI)		
			Model A†	Model B‡	Model C§
Received TIV					
2007–08	544	123	0.9 (0.7–1.1)	1.3 (1.0–1.7)	1.4 (1.0–1.9)
2008–09	564	109	0.7 (0.6–0.9)	1.1 (0.8–1.4)	1.0 (0.7–1.4)
Vaccinated with					
None	1,728	466	Referent	Referent	Referent
2007–08 only	187	51	1.0 (0.7–1.4)	1.3 (0.9–1.8)	1.3 (0.9–1.9)
2008–09 only	207	37	0.7 (0.5–1.0)	0.9 (0.6–1.3)	0.9 (0.6–1.4)
Both	357	72	0.7 (0.6–1.0)	1.3 (0.9–1.8)	1.4 (1.0–2.0)
No. TIVs in past 5 y					
None	1,450	383	Referent	Referent	Referent
At least 1	1,029	243	0.9 (0.7–1.1)	1.2 (1.0–1.5)	1.2 (1.0–1.5)
1–3	711	187	1.0 (0.8–1.2)	1.2 (0.9–1.5)	1.2 (0.9–1.5)
4–5	318	56	0.7 (0.5–0.9)	1.3 (0.9–1.9)	1.4 (1.0–2.2)
5	156	19	0.5 (0.3–0.8)	0.8 (0.4–1.4)	0.8 (0.4–1.4)
Ever received PPV23	343	41	0.4 (0.3–0.6)	0.9 (0.6–1.4)	0.9 (0.6–1.5)
Vaccinated with					
None	1,796	499	Referent	Referent	Referent
2008–09 TIV only	340	86	0.9 (0.7–1.2)	1.1 (0.8–1.4)	1.0 (0.7–1.4)
PPV23 only	119	18	0.5 (0.3–0.9)	0.9 (0.5–1.5)	0.9 (0.5–1.6)
Both	224	23	0.4 (0.2–0.6)	1.0 (0.6–1.7)	1.0 (0.5–1.8)

*TIV, inactivated trivalent influenza vaccine; PPV23, pneumococcal polysaccharide vaccine. †Unadjusted model. ‡Model adjusted for age, sex, region of residence, Socioeconomic Factor Index (*24*), and week of specimen collection. §Model adjusted for all model B variables plus no. of hospital admissions and family physician visits in previous 12 mo, pregnancy, having a chronic or immunocompromising medical condition, and antiviral drug use. Model also included mutual adjustment for 2007–09 TIV and PPV23.

In analyses limited by small numbers, study participants who received the seasonal 2007–08 and the 2008–09 TIV had a 40% increased risk for influenza A(H1N1)pdm09 virus infection compared with those who received neither vaccine. In general, there was a trend of increasing risk for influenza A(H1N1)pdm09 virus detection with the receipt of more TIVs over the preceding 5 years (Table 3). However, annual receipt of TIV over the preceding 5 years was inversely associated with the risk for pandemic virus detection. In addition, having ever received PPV23 was not associated with increased risk for influenza A(H1N1)pdm09 detection (OR 0.9, 95% CI 0.6–1.5).

There was no evidence that the association between receipt of the 2008–09 TIV and influenza A(H1N1)pdm09 detection varied by the sex, age, or place of residence of study participants; by the presence of chronic medical conditions in participants; or by the epidemic phase (Table 4). For example, the OR was 1.2 (95% CI 0.6–2.4) for participants who were >50 years of age, and the OR was 0.9 (95% CI 0.6–1.3) for younger participants (p_interaction_ = 0.31). The OR was 0.9 (95% CI 0.6–1.3) among those who did not have a chronic disease and 1.3 (95% CI 0.7–2.3) for those who did (p_interaction_ = 0.33).

**Table 4:** Effect of receipt of 2008–09 TIV on risk for infection with influenza A(H1N1)pdm09 virus, Manitoba, Canada, 2009*

Data subsets, by demographic and clinical characteristic	No. community controls, n = 2,479	No. community case-patients, n = 626	Odds ratio (95% CI)	
			Model A†	Model C‡
Sex				
F	1,468	322	0.8 (0.6–1.1)	1.0 (0.7–1.5)
M	1,011	304	0.6 (0.4–0.9)	1.1 (0.7–1.8)
p for interaction			0.304	0.997
Age group, y				
0.5–49	1,776	549	0.9 (0.7–1.2)	0.9 (0.6–1.3)
>50	703	77	1.0 (0.7–1.7)	1.2 (0.6–2.4)
p for interaction			0.563	0.308
Age, y				
0.5–4	210	62	1.1 (0.5–2.4)	1.3 (0.3–4.9)
5–19	413	223	1.1 (0.6–1.8)	1.6 (0.7–3.6)
20–34	553	154	0.9 (0.5–1.5)	0.9 (0.5–1.8)
35–49	600	110	1.0 (0.6–1.7)	0.9 (0.4–1.7)
50–59	331	56	1.4 (0.8–2.6)	1.7 (0.7–4.5)
>60	372	21	1.4 (0.6–3.5)	0.4 (0.1–3.4)
p for interaction			0.882	0.916
Locality of residence				
Rural	1,073	285	0.8 (0.5–1.1)	1.0 (0.6–1.7)
Urban	1,406	341	0.7 (0.5–0.9)	1.0 (0.7–1.5)
p for interaction			0.632	0.628
Area of residence				
North	362	156	1.0 (0.6–1.7)	1.3 (0.6–2.8)
South	2,117	470	0.7 (0.5–0.9)	0.9 (0.7–1.3)
p for interaction			0.236	0.255
Epidemic phase, 2009				
Apr 27–Jun 20	1,071	423	0.7 (0.5–0.9)	0.9 (0.6–1.4)
Jun 21–Aug 21	1,408	203	0.8 (0.5–1.1)	1.1 (0.6–1.7)
p for interaction			0.586	0.482
Chronic disease				
No	1,931	536	0.8 (0.6–1.0)	0.9 (0.6–1.3)
Yes	548	90	1.0 (0.6–1.4)	1.3 (0.7–2.3)
p for interaction			0.402	0.330
Respiratory disease				
No	1,456	386	0.7 (0.5–1.0)	0.9 (0.6–1.3)
Yes	1,023	240	0.7 (0.5–1.0)	1.3 (0.8–2.0)
p for interaction			0.831	0.764

*TIV, inactivated trivalent influenza vaccine. †Unadjusted model. ‡Model adjusted for age, sex, region of residence, Socioeconomic Factor Index (*24*), and week of specimen collection, no. hospital admissions and family physician visits in previous 12 mo, pregnancy, presence of a chronic or immunocompromising medical condition, and antiviral drug use. Model also included mutual adjustment for 2007–09 TIV and PPV23.

Among participants with laboratory-confirmed influenza A(H1N1)pdm09 virus infection, the receipt of the 2008–09 TIV was associated with a statistically nonsignificant reduction in the risk for hospitalization (OR 0.6, 95% CI 0.3–1.3) (Table 5). On the other hand, having ever received PPV23 was associated with a statistically nonsignificant increase in risk for hospitalization. However, these analyses were limited by small numbers, which resulted in wide CIs and, likely, unstable estimates.

**Table 5:** Association between receipt of seasonal influenza vaccine and subsequent risk for hospitalization among patients with influenza A(H1N1)pdm09 virus infection, Manitoba, Canada, 2009*

Variable	No. community case-patients, n = 626	No. hospitalized case-patients, n = 205	Odds ratio (95% CI)		
			Model A†	Model B‡	Model C§
Received TIV					
2007–08	123	37	0.9 (0.6–1.4)	0.6 (0.3–1.0)	0.4 (0.2–0.8)
2008–09	109	39	1.1 (0.7–1.7)	0.8 (0.5–1.3)	0.6 (0.3–1.3)
Vaccinated with					
None	466	149	Reference	Reference	Reference
2007–08 only	51	17	1.0 (0.6–1.9)	0.7 (0.3–1.4)	0.5 (0.2–1.2)
2008–09 only	37	19	1.6 (0.9–2.9)	1.3 (0.6–2.6)	0.8 (0.4–1.8)
Both	72	20	0.9 (0.5–1.5)	0.5 (0.3–1.0)	0.2 (0.1–0.5)
No. TIVs in past 5 y					
None	383	119	Reference	Reference	Reference
>1	243	86	1.1 (0.8–1.6)	0.9 (0.6–1.4)	0.8 (0.5–1.2)
1–3	187	65	1.1 (0.8–1.6)	1.0 (0.6–1.4)	0.8 (0.5–1.3)
4–5	56	21	1.2 (0.7–2.1)	0.8 (0.4–1.6)	0.5 (0.2–1.3)
5	19	9	1.5 (0.7–3.4)	1.1 (0.4–3.2)	0.6 (0.2–2.3)
Ever received PPV23	41	32	2.6 (1.6–4.3)	1.8 (1.0–3.6)	1.7 (0.8–3.8)
Vaccinated with					
None	499	153	Reference	Reference	Reference
2008–09 TIV only	86	20	0.8 (0.5–1.3)	0.6 (0.3–1.1)	0.5 (0.2–1.1)
PPV23 only	18	13	2.4 (1.1–4.9)	1.4 (0.6–3.4)	1.2 (0.4–3.1)
Both	23	19	2.7 (1.4–5.1)	1.9 (0.8–4.6)	1.6 (0.5–4.7)

*TIV, inactivated trivalent influenza vaccine; PPV23, pneumococcal polysaccharide vaccine. †Unadjusted model. ‡Model adjusted for age, sex, region of residence, Socioeconomic Factor Index (*24*), and week of specimen collection. §Model adjusted for all model B variables plus no. hospital admissions and family physician visits in previous 12 mo, pregnancy, presence of a chronic or immunocompromising medical condition, and antiviral drug use. Model also included mutual adjustment for 2007–09 TIV and PPV23.

With 626 community case-patients and 2,479 community controls and by using a 2-sided 5% significance level, our study had ≈80% power to detect an OR as small as 1.3 (30% increase in risk), assuming 19% of controls received the 2008–09 TIV (*28*). With 205 hospitalized case-patients and 640 community case-patients, the study power was considerably lower for the hospitalization analysis: the smallest detectable OR with 80% power was 1.7.

## Discussion

We found no evidence that receipt of the 2008–09 TIV increased or decreased the risk for laboratory-confirmed influenza A(H1N1)pdm09 virus infections during the first wave of the pandemic in Manitoba. In analyses limited by small numbers, the 2008–09 TIV was associated with a statistically nonsignificant reduction in the risk for hospitalization.

These results are consistent with those in the bulk of the literature. Several studies using different designs (cohort as well as test-negative and conventional case–control studies) from Australia (*4**,**5*), England (*6*), Spain (*7**,**8*), and the United States (*9**–**11*) found that the 2008–09 TIV neither increased nor decreased the risk for influenza A(H1N1)pdm09 virus infection during the first wave of the pandemic.

The lack of protective effects against influenza A(H1N1)pdm09 virus is not surprising given the substantial antigenic divergence between the pandemic virus and recently circulating seasonal influenza A (H1N1) viruses among humans (*29*) and the lack of a cross-reactive antibody response to the pandemic strain in serologic studies of TIVs for humans and animals (*12**–**14**,**30*). However, 2 case–control studies from Mexico have indicated a protective effect (35%–74%), especially against severe infections (*2**,**3*). Concerns about possible selection bias and uncontrolled confounding were raised about both studies (*31**,**32*), although a reanalysis of the second study that attempted to address these concerns confirmed the initial results (*33*). Lower levels of seroconversion among TIV-vaccinated compared with unvaccinated persons were observed among nurses in a cohort study in Canada (*15*) and among military personnel in a cohort study in Singapore (*16*). However, it is unclear whether the results from these subpopulations are applicable to the general population. In the Singapore study, TIV was not protective against seroconversion among community participants. Similar reservations might be applicable to a US case–control study that reported a protective effect for the 2008–09 TIV among active-duty military service members (*34*).

On the other hand, increased risk for influenza A(H1N1)pdm09 virus infection with receipt of the 2008–09 TIV was reported for US military beneficiaries who sought care for influenza-like illness at Navy clinics in San Diego County, California, USA, during the first wave of the pandemic (*35*). However, the positive association with confirmed subtype H1N1 infection was seen only in univariate analyses restricted to active-duty members and was not observed for other study groups. In a small pilot study from Hong Kong, 31% of children who were randomly selected to receive TIV in November 2008 had serologically confirmed influenza A(H1N1)pdm09 virus infection, compared with 12% of the children who received a placebo (*30*). However, there were no significant differences between the 2 groups in rates of influenza-like illness, acute respiratory symptoms, or PCR-confirmed pandemic infections. Four studies from Canada, including the aforementioned Ontario study, have also reported increased risk with TIV use, especially among younger persons (*1*). The inconsistency between our results and those of the Ontario study could be due to bias or residual confounding in either study.

Major strengths of our study include its population-based design and relatively large sample size. Because of the availability of accurate automated vaccination records (*23*), this study was less susceptible to recall bias and to misclassification of exposure status, issues that are common in observational studies in which vaccination information is self-reported. Misclassification of disease status was minimized by use of an accurate diagnostic test (RT-PCR) (*22*). However, it is well-known that viral RNA is occasionally not detectable by RT-PCR (e.g., because of delay in specimen collection), which means that some case-patients in our study might have been misclassified as controls. It is difficult to predict the direction of resulting bias. If the likelihood of false-negative results was not related to receipt of vaccine, our estimates would generally bias toward the null, masking any associations (*36*). If false-negative results were more likely among the unvaccinated persons (which could be the case if lack of vaccination and the delay in getting tested are caused by lack of timely access to primary care), our OR estimates could have been biased downwards, potentially masking any harmful effects of vaccination. We did not have information about testing delay, but we used proxies for access to health care (e.g., frequency of physician encounters) to adjust for factors that might be associated with promptness of testing. Stratifying the analysis by quintiles of the number of physician visits in the previous year did not result in any significant differences in the estimated ORs.

To further control for confounding by access to and propensity to seek health care, we employed a test-negative case–control design, in which all participants were tested for influenza. We believe that using test-negative controls was the most practical way to sample controls from the population that gave rise to case-patients in our study (*37*). Had we sampled from the population at large, some of these controls would have been persons who would have never been tested for influenza if they had it (e.g., because of asymptomatic infection or because of lack of timely access to ambulatory care) and would have never appeared in our database as case-patients. The resulting bias could lead to underestimation of vaccine effectiveness if, as expected, receipt of the vaccine is positively associated with better access to ambulatory care and, therefore, to testing.

The controls in our study appeared to be representative of their respective age groups in the Manitoba population, and in general, they had characteristics similar to those for the control group in the Ontario case–control study (*1*) (Table 6). For instance, in our study the percentage of controls who received the 2008–09 TIV was ≈8% for participants 12–19 years of age, 14.5% for those 20–34 years of age, 19% for those 35–44 years of age, 28% for those 45–64 years of age, and 57% for those >65 years of age (Table 6). The corresponding percentages for the Manitoba population were 14%, 13%, 18%, 28%, and 67%, respectively (*38*). In the years leading to the pandemic, influenza vaccination policy in Manitoba was consistent with the recommendations of the Canadian National Advisory Committee on Immunization (*21*).

**Table 6:** Demographic and clinical characteristics of case-patients and controls in a study of the association between the 2008–09 TIV and influenza A(H1N1)pdm09 virus infection, Manitoba, Canada, 2009*

Characteristic	No (%) study participants, by age group, y					
	<12	12–19	20–34	35–44	45–­64	>65
Community controls						
Total	381 (15.4)	242 (9.8)	553 (22.3)	384 (15.5)	638 (25.7)	281 (11.3)
Female sex	185 (48.6)	138 (57.0)	353 (63.8)	237 (61.7)	385 (60.3)	170 (60.5)
Chronic disease	30 (7.9)	21 (8.7)	57 (10.3)	74 (19.3)	203 (31.8)	163 (58.0)
Receipt of 2007–08 TIV	34 (8.9)	19 (7.9)	76 (13.7)	67 (17.4)	187 (29.3)	161 (57.3)
Receipt of 2008–09 TIV	53 (13.9)	20 (8.3)	80 (14.5)	72 (18.8)	179 (28.1)	160 (56.9)
Community case-patients						
Total	180 (28.8)	105 (16.8)	154 (24.6)	76 (12.1)	102 (16.3)	9 (1.4)
Female sex	88 (48.9)	50 (47.6)	81 (52.6)	39 (51.3)	58 (56.9)	6 (66.7)
Chronic disease	9 (5.0)	15 (14.3)	17 (11.0)	13 (17.1)	29 (28.4)	7 (77.8)
Receipt of 2007–08 TIV	26 (14.4)	9 (8.6)	24 (15.6)	21 (27.6)	35 (34.3)	8 (88.9)
Receipt of 2008–09 TIV	24 (13.3)	10 (9.5)	20 (13.0)	15 (19.7)	34 (33.3)	6 (66.7)
Hospitalized case-patients						
Total	68 (33.2)	21 (10.2)	44 (21.5)	25 (12.2)	37 (18.0)	10 (4.9)
Female sex	23 (33.8)	13 (61.9)	32 (72.7)	12 (48.0)	27 (73.0)	8 (80.0)
Chronic disease	17 (25.0)	5 (23.8)	10 (22.7)	13 (52.0)	25 (67.6)	8 (80.0)
Receipt of 2007–08 TIV	6 (8.8)	0 (0.0)	4 (9.1)	9 (36.0)	12 (32.4)	6 (60.0)
Receipt of 2008–09 TIV	9 (13.2)	3 (14.3)	4 (9.1)	5 (20.0)	14 (37.8)	4 (40.0)

*TIV, inactivated trivalent influenza vaccine.

Information about several confounders was obtained from administrative databases. The completeness and accuracy of the MH database are well established, and these databases have been used extensively in studies of postmarketing surveillance of various drugs and vaccines (*39*). However, it is possible that there was a measurement error in some variables, which could result in residual confounding. In addition, the protective effects we observed against hospitalization might be related to confounding by factors that were not measured in this study, e.g., functional capacity (healthy vaccinee bias) (*40*).

Results from our study in Manitoba corroborate findings from studies outside Canada that the 2008–09 TIV neither increased nor decreased the risk for influenza A(H1N1)pdm09 virus infection. Additional epidemiologic and experimental investigations are needed to clarify the relationship between TIV use and infection with the pandemic strain.
